# Nutritional Habits in Crohn’s Disease Onset and Management

**DOI:** 10.3390/nu17030559

**Published:** 2025-01-31

**Authors:** Konstantinos Papadimitriou, Georgia-Eirini Deligiannidou, Gavriela Voulgaridou, Constantinos Giaginis, Sousana K. Papadopoulou

**Affiliations:** 1 Faculty of Sport Sciences & Physical Education, Metropolitan College, University of East London, 54624 Thessaloniki, Greece; 2 Department of Nutritional Sciences and Dietetics, School of Health Sciences, International Hellenic University, 57001 Thessaloniki, Greece; deligiannidoueirini@yahoo.gr (G.-E.D.); gabivoulg@gmail.com (G.V.); souzpapa@gmail.com (S.K.P.); 3 Department of Medicine, School of Health Sciences, Democritus University of Thrace, 68100 Alexandroupolis, Greece; 4 Department of Food Science and Nutrition, School of Environment, University of Aegean, 81100 Myrina, Greece; cgiaginis@aegean.gr

**Keywords:** inflammatory bowel disease, Crohn’s disease, diet, intestine, symptoms

## Abstract

Crohn’s disease (CD)’s activation factors are still unclear. However, they are reported to involve an interaction between genetic susceptibility and unhealthy lifestyle factors like smoking, alcohol consumption, low physical activity, low BMI (<18.5 kg/m^2^), and probably unbalanced nutritional habits. Therefore, the aim of the present review is to demonstrate the possible effects of different nutritional habits, before the occurrence of the disease, as crucial factors for the inception of CD activation. The structure of the present narrative review was conducted following the instructions of the “Review Academy of Nutrition and Dietetics Checklist”. It is well established that the consumption of specific foods and drinks, such as spicy and fatty foods, raw vegetables and fruits, dairy products, carbonated beverages, and coffee or tea, can provoke the exacerbation of CD symptoms. On the other hand, Mediterranean-oriented diets seem to provide an inverse association with the incidence of CD. Moreover, patients seem to have the knowledge to select foods that contribute to the remission of their symptoms. However, it is not clearly reported whether the onset of CD activation is due to lifelong unbalanced nutritional habits and their subsequent effect on gut microbiota secretion, which seems to be the gold standard for CD’s investigation. Therefore, more future studies should record, examine, and compare the nutritional habits between patients with CD (immediately after the disease’s diagnosis) and healthy populations in a lifelong manner, in order to reveal the possible influence of foods on CD onset.

## 1. Introduction

In 1932, Burrill B. Crohn first published his report on a disease that mainly affects young adults, causing inflammation in the intestine and specifically the ileum [1]. Nowadays, inflammatory bowel disease (IBD) is known as a chronic inflammatory disease which is categorized into Crohn’s disease (CD) and Ulcerative colitis (UC) [2]. The difference between the two lies in where the inflammation is primarily concentrated: in UC, the colon is predominantly affected, whereas in CD, inflammation can occur anywhere along the intestinal tract. Despite the focused locations and lesion patterns, CD also demonstrates more intensive symptoms [3] than UC. Recent demographic studies from 2000 to 2020 reported that the incidence and prevalence of CD in Asian [4] and European countries [5] range from 0.34 to 3.91, and 8 to 13, respectively, while mixed IBD incidence and prevalence statistics (both CD and UC cases) for North American and Canadian citizens showed 286 and 319 cases per 100,000 inhabitants, respectively [6].

A variety of symptoms may be present in the onset and development of CD. Such symptoms may vary between patients, and most commonly include diarrhea, abdominal pain, fatigue, weakness, anorexia, and malnutrition, often leading to alterations in patients’ body composition [7]. In addition, particularly in cases of severe conditions, symptoms of chronic anemia, abnormal development, bloody diarrhea, toxic megacolon, and the development of mesenteric white adipose tissue (mWAT) hypertrophy are also observed [2,8,9]. Other CD manifestations include skin lesions, arthritis, osteoporosis, and eye and liver disorders [9]. It should be highlighted that there is a psychological burden in IBD, which in the cases of patients with CD can manifest as high levels of depression and anxiety according to previous observations [2,10].

To the best of our knowledge, the factors that activate CD in young, middle aged, and elderly people [11] are still unclear. Although the core characteristic of the disease is inflammation (either chronic or reoccurring), its onset could be attributed to an interaction between genetic susceptibility and various unhealthy lifestyle factors like smoking, alcohol consumption, low physical activity [12,13], low BMI (<18.5 kg/m^2^), and probably unhealthy nutritional habits, which are mainly related to Westernized diets and the consumption of ultra-processed foods [12,14].

Individuals’ overall nutritional status and specific food patterns can yield either positive or negative outcomes on the immune system [15]. Unhealthy nutritional habits are usually related to the perpetual consumption of ultra-processed foods and beverages (e.g., fat-rich foods) [15], the relation of which to the immune defense response has already been highlighted [16]. Additionally, nutrition plays an essential role in the gut’s microbial functioning and composition, host immunity, and intestinal physiology and barrier [17]. Specifically, gut microbiomes are adapted to changes in food patterns, while macronutrients regulate the levels of beneficial and harmful microbes, toxic microbial metabolites, and protective metabolites [18]. It must be noted that micronutrients such as vitamins, minerals, and non-nutritive bioactive compounds play a crucial part at various levels of innate immune response, while their coadjuvants in several stages are essential in order to maintain optimum responses and an immune system that is adaptive to the gut microbiome. In this setting, the deficiency of even one nutrient can impair immunity, causing significant immunodeficiency with clinical repercussions, and thus chronic undernutrition and nutrient deficiency affect immune cell circulation and cytokine response [17].

On the other hand, the excessive intake of some micronutrients can also impair immune responses. Although conflicting evidence exists, there is research supporting the theory that supplementations with vitamin D and zinc may mitigate excessive immune responses [18]. Particularly in the case of vitamin D, this may also be related to vitamin D receptor (VDR) deficiency.

In the context of evaluating imbalances in dietary patterns and their effects on the inflammation levels of the body, it must be made clear that abnormal body fat storage increases inflammatory markers in systemic trafficking, resulting in chronic low-grade inflammation [16]. As such, obesity is associated with an elevated postprandial inflammatory response [19], and patients with increased abdominal fat mass had elevated inflammatory cytokines, prothrombotic molecules, and adhesion molecules [20]. Notably, in Westernized society, there is a rising rate of immune-mediated diseases. Although there is compelling evidence that healthy diet patterns, such as the Mediterranean diet, with components like vegetables, fruits, whole grains, and fatty fish rich in omega−3 fatty acids, are associated with lower inflammation [21] as well as a positive impact on the microbial ecosystem [22], the observed shift of the population toward lower adherence [23] to such patterns is alarming.

Within this framework, it is clear that although the specific nutritional approaches [24] are still under study, dietary management of the disease is a key element from the onset and throughout its development. Therefore, the present review aims to demonstrate the possible effects of different food habits and their excessive consumption as central contributors to CD onset.

## 2. Materials and Methods

### 2.1. Detailed Analysis of the Review Structure

The structure of the present review was conducted following the instructions of the “Review Academy of Nutrition and Dietetics Checklist” [25]. Thus, the manuscript contains the following:

1. Title: Specific, and noting that this is a review.
2. Abstract content: (a) Background, (b) objectives, (c) brief summary of re-view, and (d) implications for future research.
3. Introduction: (a) General information about the topic, (b) key questions which are analyzed in the “Discussion” section.
4. Methods: (a) Detailed analysis of the review’s structure, (b) study selection (inclusion and exclusion criteria: publication year, language, type of article, and databases).
5. Discussion: (a) Answers on the key questions, (b) possible limitations from the literature to answer the key questions.
6. Conclusions: (a) Brief summary of the findings, (b) future studies to be conducted.

### 2.2. Studies Selection

The studies were selected according to the following inclusion and exclusion criteria. Table 1 shows the requirements utilized for the studies’ selection and a comment about the reason for this selection [20].

The combinations of terms which were used was “Dietary patterns cause Crohn’s disease development”; “Diet activates Crohn’s disease”, “Inflammatory bowel disease’s causes”, and “Crohn’s disease nutrition mechanisms”. Also, on the PubMed MEDLINE database, filters for the publication date (from 2010 to 2024) and for the types of article (clinical trial and randomized controlled trial) were used. In addition, only the articles which could answer the main topic of the review were analyzed. Therefore, 61 studies were used: 25 for the introduction and methodology sections and 36 for the discussion section.

## 3. Discussion

### 3.1. Foods, Beverages, and Diets: The Good and the Bad in CD

As previous studies have focused on patients’ nutritional habits, in an effort to shed light upon the contribution of foods and beverages that may be key modifiers of the exacerbation or remission of CD symptoms [26], a vast heterogenicity in the studies and populations is currently leading to a lack of consensus on individualized dietary recommendations [27]. An essential remark regarding the overall view of current research is that there is no association between a priori or a posteriori dietary patterns for CD risk [28]. As a result, in their studies, authors provide general instructions and observations about the effects of foods and beverages on CD symptoms.

Starting with a recently documented case study [12], it has been reported that an athlete with no documented genetic predisposition and inadequate nutrition intake in both macro and micronutrients manifested CD symptoms after consuming a slice of pizza. This incidence may indicate that insufficient food consumption can probably activate CD symptoms, and nothing short of a single meal can be the final drop that will trigger the disease’s onset. Although this study is not a basis from which to draw conclusions, it is an important remark on the limitations related to the proper and timely evaluation of CD. This is extremely relevant to the underreporting of case studies, as well as the lack of a central documentation system of dietary history records alongside the medical records of the patient.

Focusing on larger-scale studies, Cohen et al. [26] recorded the daily nutritional habits in a total of 2329 Americans, in an attempt to identify foods that exacerbate or reduce IBD symptoms. From the results, patients with CD prefer foods such as yogurt, rice, and bananas, which were reported to inhibit symptoms’ manifestation. On the other hand, foods such as leafy and non-leafy vegetables, spicy foods, fruits, nuts, fried foods, milk, beans, soda, popcorn, dairy, alcohol, seeds, coffee, red meat, fatty foods, and food with high fiber content seem to exacerbate CD symptoms [26]. These observations are not very dissimilar to what current dietary interventions for the management of the disease often look like, and are highly relevant to what is demonstrated by the empirical evidence, namely, the exclusion of several foods by patients in order to avoid flare-ups of symptoms. However, in CD ostomy patients, the authors found a higher consumption of cheese, sweetened beverages, milk, pizza, and processed meats compared to patients with CD [26]. As such, the observations may hint at the flexibility related to ostomy use in patients with CD, which is also related to studies investigating the effects of exclusive enteral nutrition (EEN) [27], particularly in CD.

It is usually observed that patients with CD who have any past-surgery condition [26] or are in remission due to a biological agent (Anti-Tumor-Necrosis-Factor-a (Anti-TNFα)), in both responders and non-responders to the treatment, have similar caloric intake from ultra-processed foods [29], independent of the number of ingredients having negative effects on the intestinal microbiome [29]. Such variations may be related to the observation of many patients with CD excluding foods from their diet because of the concern of a flare-up of the symptoms [26], but also to the different tolerance levels that patients may exhibit to the consumption of specific foods. Also, exogenous and psychological factors are key players given these differences.

A simple research hypothesis considers that if CD manifestation and exacerbation are dependent on poor nutrition intake, such as that found in Westernized diets [6] which consist of a high consumption of fats and sugars and probably cause gut dysbiosis [30], then a balanced diet such as the Mediterranean diet (MD), which is based on plant foods, mainly cereals, vegetables, and fruit, as well as olive oil and small portions of dairy products, sweets, sugar, and meat, could potentially protect against, remiss, or reduce the incidence of CD symptoms [31].

This is in fact an aspect that has gained large scientific interest; however, the results of MD are often contradictory. Such findings are often due to many patients showing gastrointestinal symptoms [32], and many foods in the MD are innately difficult to digest [30]. As Gkikas et al. [27] noted, it is possible that the choice of food consumption must vary depending on the condition of the disease. Thus, the protection, management, and remission of CD may require a different dietary approach. As described in a recent article on the subject, a personalized pattern based on the principles of MD could very well be an approach that will benefit the patient [33].

Another extensively discussed food, in the framework of healthy diets in general but also particularly in the case of IBD, is red and processed meat. In this setting, Albenberg et al. [34] reported insufficient evidence in order to recommend a reduction in red and processed meat consumption, avoiding a possible symptomatic relapse among American patients with CD. Specifically, patients with CD in remission (while on medication), in a randomized trial, showed no significant differences in disease relapse, according to the frequency of meat consumption [low (≤1 serving/week) vs. high (≥2 servings/week)] groups [34].

What is highly relevant in the case of meat consumption is perhaps side dishes, which may include sauces, the type of meat consumed (related to the fat content), the preparation method, and also the possibility of alcohol consumption. Such factors would have been eliminated in the setting of a clinical trial, and as such the key remark of this study is perhaps mostly related to the stage of the disease (i.e., remission (CD activity index (sCDAI) scores of 150 or less)), in which case it is not rare to observe better tolerance in dietary alterations. It should be noted that the literature highlights that there is in fact a link between meat consumption and all-cause mortality in patients with IBD, and as such these findings ought to be viewed with caution [35].

The self-reporting of dietary habits is a method often used in research. The formulation of a questionnaire that will also include the beliefs of the patients regarding their health and dietary status is also relevant in the case of IBD. As such, a 2016 study involving British patients with IBD by Limdi et al. [36] examined the dietary behaviors and beliefs of patients attending IBD clinics. According to this study’s results, spicy food (41.1%), fatty food (29%), alcohol (21%), raw vegetables and fruits (19%), dairy products (16%) carbonated beverages (12%), coffee or tea (11%), and sugary food (10%) were reported to worsen IBD symptoms. It is important to note that almost half of the participants never received any formal dietary advice, while the majority (over 60%) deprived themselves of their favorite foods to prevent relapse. Additionally, the vast majority of the participants believed that their active IBD affected their appetite, a trait that is even more obvious during a relapse.

Similar outcomes concerning the dietary patterns of patients with CD were shown by Zallot et al. [37] in a study investigating the nutritional facts, beliefs, and behaviors of French patients with IBD and the impact on their social life. Out of 244 participants, 177 (72.5%) were patients with CD, compared with 67 (27.5%) who were patients with UC. All of the participants were undergoing medication support with mesalamine, systemic steroids, immunomodulators, and anti-TNFα. Notably, a drawback of this study was that the food patterns were not analyzed according to the IBD category (CD and UC), which also highlights a limitation for other studies as well. However, as this study reports, the majority of the subjects shared the belief that specific foods can provoke an exacerbation of IBD symptoms (57.8%). For this reason, they reported that they prefer to avoid several foods, such as spicy food (80.7%), fat (48.8%), raw vegetables and fruits (47.5 and 45.1%), fiber (40.6%), carbonated beverages (27.5%), dairy products (43.9%), and coffee or tea (26.2%). As a result, and closely related to the psychological and social burden of the disease, the majority of the patients have reported feeling uncomfortable having dinner in a restaurant, and have also expressed the need for professional advice regarding the appropriate diet that they should follow [37].

Closely aligned to the commentary on the previous studies, as well as that in the introduction of this article, it is clear that a vicious cycle connects the triggers that onset and maintain the disease. This can happen through either poor dietary choices that lead to inflammation and malnutrition and can cause changes in body composition that can cascade to hormonal imbalances and hence trigger psychological implications, or due to psychological and social burdens that can lead to isolation and limit access to proper nutritional and medical consulting, and hence poor dietary choices. In this setting, nutritional education for patients and health care providers alike is essential. It can perhaps be linked to the following segment of this review, focusing on the nutritional value of foods. The following table (Table 2) presents an overview of the study findings related to specific foods.

Patients with CD consume foods that may affect the disease’s symptoms positively or negatively. However, it is important to examine the effects of Mediterranean, Western, and high-carbohydrate diets, among others.

Chiba et al. [30] assessed the effects of a semi-vegetarian diet (SVD) on preventing a possible relapse of CD symptoms. Specifically, the SVD was designed to raise the level of beneficial bacteria through a plant-based diet that contains foods such as green tea, brown rice, and prebiotics. However, in the diet program, fish and meat were added once a week and twice weekly, respectively. The Japanese patients’ daily food consumption was 1700 kcal/day, containing all the macro and micronutrients necessary for a balanced diet. In addition, patients received medication (infliximab and sulfasalazine).

According to the results, SVD was found to be safe and effective in the remission of CD symptoms [30]. Therefore, compared with remission, patients had a normal CRP concentration at the final visit to the hospital. Notably, the overwhelming majority (15/16) of patients who continued to follow the SVD at 2 years follow-up were free from relapse and the rate of remission was 100% and 92% after 1 and 2 years of the intervention, respectively. On the other hand, in the omnivorous group, the remission rate was only 67% and 25% after 1 and 2 years of the intervention, respectively. It is probable that the medication influenced the symptoms’ remission. However, in Papadimitriou’s study [12], in which the patient received medication such as Metronidazole, Mesalazine, Ciprofloxacin, and Salofalk, the symptoms were exacerbated. Therefore, a balanced diet could be a crucial factor for medical effectiveness.

Kang et al. [40] studied the effectiveness of a short-term partial enteral nutrition (SPEN) diet containing 14% protein, 54.5% carbohydrate, and 31.5% fat and providing 20 kcal/Kg body weight in Korean pediatric patients with CD with severe symptoms. According to the results, patients’ nutrition status after one year of intervention was improved, while the severity of their symptoms was moderated.

Specifically, hematological indexes of Hemoglobin, Transferrin, Ferritin, Prealbumin, Albumin, Zinc, Calcium, Magnesium, Vitamin A, Vitamin B_12_, Vitamin E, and Folate were improved after the intervention. Moreover, body weight and pediatric Crohn’s disease activity index (PCDAI) were also improved in the SPEN group compared to the non-SPEN group. However, 25-OH-vitamin D levels were not improved in the SPEN group, probably due to the low consumption of foods containing vitamin D, or another issue which must be examined [40].

Another diet which has been reported as beneficial is the carbohydrate diet. A specific carbohydrate type of diet (SCD), which eliminates all grains and sugars (except for honey) as well as all dairy products (except for yogurt and hard cheeses) and all processed foods, seems to be a promising method for the control of IBD symptoms, especially in CD [41].

Suskind et al. [41] studied the effects and the clinical impact of the SCD, a modified SCD with oats and rice (MSCD), and a whole food diet (WF) in young American patients with CD. In the study, through the 12-week intervention, at three time points (baseline, 2nd week, and 12th week), the metabolomics, metagenomics, and proteomics were measured. In addition, clinical measurements of complete blood count (CBC), CRP, erythrocyte sedimentation rate (ESR), calprotectin (baseline and 12th week), and the PCDAI were also performed at the same three time points of the intervention [41].

According to the results, through the PCDAI, the three types of diet demonstrated an improved clinical image. However, the two carbohydrate diets had greater improvements for CD remission, affecting the ESR and CRP positively in comparison with the whole food diet. Also, in the metabolomics, metagenomics, and proteomics analysis, no difference was observed in metabolic pathways among the different diets. Nevertheless, the authors considered that it is difficult to demonstrate any statement because of the small number of participants. Last but not least, calprotectin did not demonstrate any strong alteration, and instead it was reduced in the WF and MSCD groups [41].

Regarding nutritional interventions, the Mediterranean dietary pattern, which is rich in beneficial components such as vitamins, minerals, dietary fibers, and antioxidant and anti-inflammatory actor, seems to have the potential to maintain a healthy and diverse gut microbiota, and improve and modulate the immune system.

The Mediterranean Diet (MD) emphasizes the consumption of whole grains, seeds, nuts, legumes, vegetables, and fruits, followed by a moderate consumption of fish, poultry, and dairy products. Also, it focuses on extra virgin olive oil as the principal source of fat, while processed red meat products and sugars should be low [42]. A healthy lifestyle, based on the MD, accompanied by sufficient physical activity and adequate sleep and rest and combined with the successful management of stress and anxiety, is vital for a strong immune system and lower levels of inflammatory markers such as CRP, calprotectin, TNFα, IL17, IL 12, and IL13 [42,43].

Racine et al. [28] studied the possible associations between the appearance of UC and CD and dietary patterns in European counties (Denmark, France, Germany, Italy, The Netherlands, Sweden, and the United Kingdom). A famous dietary pattern which is mostly followed in European countries is the MD, which is characterized by a high consumption of vegetables, legumes, fruits and nuts, cereal products, fish, olive oil, and wine, and a moderate-to-low consumption of meat and dairy products. Many foods proposed by the MD were selected by patients with IBD for the remission of CD symptoms [20].

Thus, in a large European prospective study, from a sub-cohort of 366,351 participants, 256 and 117 were diagnosed with UC and CD, respectively. According to the results, patients with CD (32 men and 85 women, with a median age of diagnosis around 50 years), had a weak compliance with the adapted Mediterranean diet (aMED). More specifically, a low consumption of vegetables in combination with a high consumption of sugar and soft drinks were associated only with UC onset. Further studies are necessary in order to explore whether other mechanisms such as microbiota alterations could mediate this association [28] (Table 3).

### 3.2. Nutritional Value of Foods

Taylor et al. [45] found that Canadian patients with CD, compared to the representative sample of healthy individuals, had similar macronutrient but different micronutrient intakes, with patients with CD having a significantly lower intake of vitamins C and D, thiamin, niacin, magnesium, phosphorus, zinc, and potassium. In addition, PUFA (Omega 3 and 6) and MUFA intakes were significantly lower in patients with CD [38,39].

On the other hand, neither total fat, n-3 PUFA, n-6 PUFA, oleic acid, arachidonic acid, or linoleic acid intake were associated with CD severity, as depicted by Ananthakrishnan et al. [39]. In this study, the participants’ ages [39] could be considered a methodological limitation due to the fact that in older patients, dietary habits tend to be more difficult to modify due to their long-term adherence to these practices. This factor reveals two issues. Firstly, it is difficult for an adult, especially an older adult, to consume foods that could potentially have a beneficial effect on the symptoms of CD, due to the concern of a possible worsening of the disease [39], an observation that is significantly related to a lack of nutritional education throughout their lives or perhaps the limited construction of trust between the patient and the health care provider. Secondly, there is a percentage of patients who consume foods that are forbidden considering the disease’s condition [45]; however, they have developed a tolerance to their consumption, and thus they are not directly linked to symptom manifestation for these particular patients [35].

On the other hand, Lee et al. [46], Labriola et al. [47], and Hartman et al. [48] recorded the food habits of pediatric CD cohorts. Lee et al. [46] revealed a total of 1325 unique foods that contain additives such as xanthan gum, carrageenan, maltodextrin, and soy lecithin. The recordings were conducted according to the foods that the participants were buying from one of the largest food and drug grocery stores in the U.S. According to the study’s results, children with CD frequently consume food additives, probably associated with intestinal inflammation and CD symptoms [45].

Similarly, Labriola et al. [46] examined the food intake of 68 Italian CD subjects aged 4 to 18 years old and found similar results to studies involving adult patients with CD [20,36]. Specifically, pediatric patients with CD consumed lower amounts of fiber, omega 3 fatty acids, vitamin A, beta-carotene, and polyphenols, and higher amounts of animal proteins, niacin, and vitamin B12. Additionally, no differences were recorded in carbohydrate and other macro- and micronutrient consumption in these dietary patterns, which have been described as not dissimilar to the so-called Western diet. Notably, given the young age of the participants in this study, the food choices recorded could also be involved in CD pathogenesis. However, the lack of a control group [34] in this study is a limitation, and one observed in several trials, thus making it difficult to either draw solid conclusions or to make comparisons with relevant trials.

Also, in the same setting, Hartman et al. [48] investigated the dietary habits of pediatric patients with IBD from Israel. The outcomes of this study demonstrated that the patients had significantly poor carbohydrate, calcium, magnesium, vitamin A and E, and fiber intake, while documenting higher intakes of protein, iron, and water-soluble vitamins.

According to the context of the aforementioned studies, there is a link between dietary habits and specific food selection, which is yet unclear but worth exploring. As previous studies in the setting of dietary habits have demonstrated, there is more to adhering to a pattern than following the guidelines of food groups [49]. Particular interest must be focused on the nutrients provided by each food, which is also highly related to their impact on the microbial ecosystem of the patient. Also, the limitation of a dietary record of the patient is underlined here as well, particularly in the case of younger patients and subjects who were healthy before CD occurrence. On that note, and looking further into the future strategies for CD management, such records could also be relevant in the identification of epigenetic factors of disease onset, linking the dietary choices of the parents to the risk of onset in the offspring.

### 3.3. The Role of the Gut Microbiota in CD Onset

Patients with CD showed a lower microbial α-diversity, richness, and evenness, and produced several groups of bacteria that were unstable compared with those of healthy individuals [50]. Also, they showed alterations in the composition of microbiota in the intestinal tract, which were due to the type, the stage of the disease [51], and the age at manifestation [52]. Buffet et al. [53] found that the stage of the disease differentiates the gut microbiota. Specifically, in a novel study, CD was categorized into three groups of microbiota profiles: G1, which is similar to normobiosis, and G2 and G3, which display some kind of dysbiosis with increased values in *Proteobacteria*.

Specifically, the transition from normobiosis to dysbiosis (G1 to G2) showed the decrement of anti-inflammatory bacteria such as *Roseburia*, *Eubacterium*, *Subdoligranumum,* and *Ruminococcus* and the increment of pro-inflammatory bacteria such as *Proteus* and *Finegoldia* [54]. Similarly, early-stage Chinese patients with CD showed a reduction in a variety of short-chain fatty acid (SCFA)-producing bacteria such as *Blautia*, *Clostridium IV*, *Coprococcus*, *Dorea*, and *Fusicatenibacter,* and an increment in *Escherichia/Shigella* and *Proteus* [54].

Meanwhile, the transition from G2 to G3 shows a further worsening of the symptoms’ severity, and manifests an increment in pro-inflammatory bacteria such as *Klebsiella, Pseudomonas, Salmonella*, *Acinetobacter*, *Hafnia*, *Staphylococcus*, *Enterococcus*, and *Streptococcus*. Also, Ma et al. [54] recorded the enriched presence of *Parabacteroides*, *Bacteroides*, *Escherichia/Shigella*, and *Proteus*. All these microbiotas are regarded as coinciding with the disease manifestation.

Similarly, calprotectin examination of a Belgian population with CD and a healthy cohort (HC) from the UK revealed that the concentration of intestinal microorganisms between patients with CD and healthy controls (HC) differentiates. Specifically, an increased presence of Escherichia and Fusobacterium and a slight rise in *Collinsella* was observed, in contrast with HC. On the other hand, patients with CD showed lower concentrations in anti-inflammatory microbiota such as *Peptostreptococcaceae*, *Anaerostipes*, *Christensenellaceae*, and *Methanobrevibacte* [50].

In a US cohort with pediatric Crohn’s disease (12.4 years old), the well-known microbiota of *Clostridiales*, *Pasteurellaceae*, *Veillonellaceae*, *Erysipelotrichaceae*, and *Bacteroidales* were identified, along with the newer *Campylobacter*, *Akkermansia*, *Collinsella*, and *Desulfovibrio*. However, the largest increase was identified for *Aggregatibacter*, and the greatest decrease was shown for *Roseburia* [52]. Gut microbiota excretion is associated with CD manifestation, although this is a research topic that must be further elucidated. Its elucidation depends on factors which affect gut microbiota secretion. The present review focuses on macronutrient intake, which has a 57% influence on the concentration of gut microbiota. However, genetic predisposition remains a strongly correlated factor (12%) for the disease’s manifestation [55].

The development of microbiome modulators such as diet alters the composition of the host microbiota, replacing some of the defective microbes. Therefore, the type of diet can affect CD symptoms [42,56,57,58]. According to Shanahan et al. [57], food patterns modulate the composition of intestinal microbiota. A modification of the Western diet provoked clinical improvement and a decrease in inflammatory burden in CD, and a high-fat diet also altered the composition of both positive and negative bacteria such as *Bifidobacterium*, *Eubacterium rectale–Clostridium coccoides*, and *Bacteroides*, respectively [49]. A Mediterranean diet rich in omega-3 fatty acids, vitamins, minerals, phytonutrients, and polyphenols could help in the development and maintenance of a strong immune system and a healthy and diverse gut microbiota, reducing the risk of infections and inflammation [42].

Carbohydrate diets, containing both simple and complex carbohydrates, are recognized as an important food component and a main energy source with an effect on blood glucose levels [42,58]. Several studies have proven that different kinds of carbohydrate consumed interact with the small intestine differently, causing alterations in gut microbiota [59]. Specifically, wholegrain wheat possibly increases the levels of *Lactobacillus* and *Bifidobacterium*, whereas corn may increase the levels of *Bifidobacterium*. Also, wholegrain barley may increase the levels of *Firmicutes*, particularly of the *Blautia* genus [59]. *Fructo-oligosaccharides* (FOS) occur in many fruits and vegetables and can be made in a lab as prebiotics. However, as the authors reported, the small sample sizes and an extended microbiota analysis might demonstrate increases or alterations in the concentration of the non-targeted bacteria [59].

Also, in many studies, it is regarded that the use of probiotics, prebiotics, and postbiotics provokes a great preventive response in microbiota modulation, providing various therapeutic effects [60]. Specifically, multistrain probiotics based on specific genera of *Lactobacillus* and *Bifidobacterium* showed significant efficacy in endoscopic remission and clinical relapse, and ranked best in UC. In CD, synbiotics comprising *Bifidobacterium* and a fructo-oligosaccharide/inulin mix and *Saccharomyces* ranked best in improving clinical remission and reducing clinical relapse, respectively, compared to a placebo [61]. In conclusion, probiotics, prebiotics, and postbiotics are a well-studied topic with great potential for IBD management.

Generally, patients with CD show an altered microbiome composition and a more unstable microbial community [38]. According to Pascal et al. [50], microorganisms including *Faecalibacterium*, *Peptostreptococcaceae*, *Anaerostipes*, *Methanobrevibacter*, *Christensenellaceae*, *Collinsella*, *Fusobacterium*, and *Escherichia* have been identified in the small intestine of patients with CD in different compositions compared to healthy cohorts [50].

Therefore, microbiome alteration can be a predictive factor for CD [56], while unbalanced nutritional habits provoke alterations in microbiome composition [56]. Thus, children with unbalanced macro- and micronutrient diets may have an increased risk for CD activation. However, future research and systematic reviews ensuring the integrity of the included studies will enhance this scientific topic.

## 4. Conclusions

**Figure 1:**
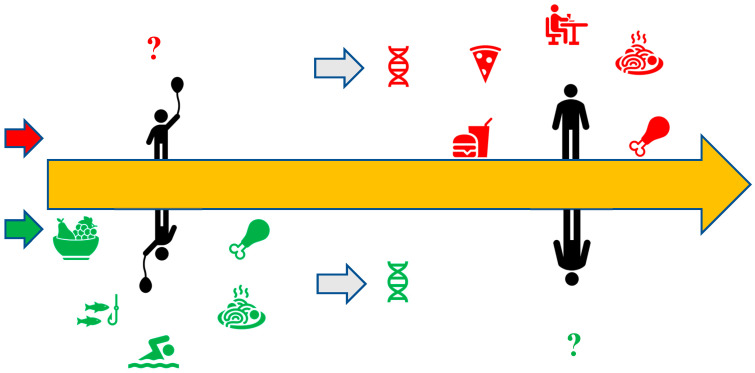
The two dimensions of nutritional habits, from a young age to adulthood, and their lifelong effects. 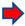 According to the literature, high-fat and unhealthy dietary habits can affect CD activation and exacerbation. **?** However, it is not well established whether CD activation is due to unhealthy dietary habits adopted from a young age. 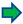 According to the literature, balanced diets contribute to the disease’s symptom remission. **?** However, it is not well established whether a healthy dietary pattern followed from a young age can decrease the possibility of activating CD in adulthood. 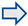 Nutrition effects on a molecular level, probably through specific gut microbiota secretion. Differences are observed between CD and healthy cohorts.

Patients with CD follow balanced diets, such as Mediterranean or semi-vegetarian diets, and prefer foods like olive oil, fish, poultry, and vegetables in an attempt to improve their quality of life by reducing intestine inflammation. Therefore, implementing a balanced diet as early as possible as a nutritional habit can be a key modifying factor for the avoidance of CD activation because of the controlled secretion of the gut microbiota, reducing the risk of infections and inflammation. This is also related to constant nutritional education of the population, as well as flexibility of health care professionals such as dieticians and gastroenterologists, in order to provide alternatives tailored to patients’ needs, as well as cultural and socioeconomic characteristics. If this principle is maintained, then individuals may have a better chance of reducing the risk of CD onset. On the other hand, an unbalanced diet that is adopted from childhood can increase the risk of CD manifestation without a genetic predisposition being a prerequisite. The gut microbiota’s harmful secretion via high-fat and unbalanced diets is suggested to contribute to the disease’s activation. Moreover, the management of the disease is still rooted in the education of the patient and a shift towards healthier food choices as well as lifestyle changes, without discarding the psychological burden, and hence the patient may need support while taking steps to improve their health. Therefore, the contribution of different foods and diets to the gut microbiota seems to be the gold standard for the next research steps (Figure 1). Therefore, longitudinal studies assessing the influence of early dietary interventions on long-term CD outcomes, recording daily food consumption, and considering the gut microbiota concentration concerning questionnaires are proposed for clearer statements and conclusions on this topic. Also, along with artificial intelligence (AI) technology, emerging digital tools (e.g., apps for dietary tracking) could guide users, avoiding any unhealthy nutritional habits and alerting cohorts about the possibility of showing IBD symptoms in the future.

## Figures and Tables

**Table 1:** Inclusion and exclusion criteria and comments about the articles chosen.

Criteria	Inclusion	Exclusion	Comment	Limitations
Publication year	After 2010	Before 2010	Use of the most recent literature	Probable exclusion of crucial studies
Language	English	No English	Understanding of studies	-
Types of article	Original articles, reviews, and case reports	Opinion studies or letters to the editor	Precise content analysis for each study	Different study designs that bias the generalization of the results
Databases	Google Scholar PubMed-MEDLINE	Other databases	Collecting studies from the most valid databases and high-impact journals	-

**Table 2:** Foods and beverages that cause remission and exacerbation and are avoided by patients with CD.

Nutritional Habits					
Remission		Exacerbation		What Patients with CD Avoid	
Foods	Beverages	Foods	Beverages	Foods	Beverages
Yogurt [26]	Kefir [38]	Vegetables [25,28]	Milk [26]	Fruit [34,37]	Milk [34]
Bananas [26]	Dairy products [39]	Spicy foods [26,36]	Soda [26]	Vegetables [34,37]	Carbonated beverages [37]
Rice [26]	Wine [39]	Nuts [26]	Alcohol [26,36]	Popcorn [34]	Coffee [37]
Olive oil [38,39]		Beans [26]	Coffee [26,36]	Ice cream [34]	Tea [37]
Potatoes [39]		Popcorn [26]	Carbonated beverages [36]	Processed meats [34,37]	Dairy products [37]
Fish [39]		Fried foods [26]	Tea [36]	Desserts [34]	
Chicken [39]		High-fiber foods [26]		Tomatoes [34]	
Vegetables [39]		Red meat [26]		Dairy products [34]	
Fruits [39]		Processed meat [34]		Pizza [27]	
Meat [39]		Dairy products [36]		Spicy food [37]	
		Fruits [36]		Fibers [30]	
		Butter [36]			

**Table 3:** Different types of diets and CD.

Authors	Type of Diet	Diet Content	Participants	Results
Racine [28]	Mediterranean Diet (MD)	Vegetables, legumes, fruits and nuts, cereal products, fish, olive oil, wine, and moderate-to-low consumption of meat and dairy products	32♂–85♀ 50 years old	Weak compliance with Mediterranean diet
Chiba [30]	Semi-Vegetarian Diet (SVD)	Plant-based	14♂–8♀ 19–77 years old Remission	High remission rate: 1st year: 100% 2nd year: 92%
Suskind [32]	Carbohydrate	SCD, MSCD: oats and rice WF: eliminating wheat, corn, sugar, milk, and food additives	8♂–6♀ 7–18 years old 4 SCD–4 MSCD–3 WF Mild to moderate symptoms	Positive effects on CD symptoms from each diet type
Kang [40]	Short-term Partial Enteral Nutrition (SPEN)	Balanced	34♂ <13 and ≥13 years old 17 SPEN–17 no SPEN Severe	SPEN group improved nutrition status after 1 year
Svolos [44]	Individualized Food-Based Diet	Excluded: gluten, lactose, and alcohol Included: macronutrients, vitamins, minerals, and fiber	Children: 5 participants 6–15 years old Relapsed in CD Adults: 12♂–13♀ >18 years old Rats: Heterozygous HLA-B27 and HLA-B7 36–40-week-old	60% of the children entered in remission and decreased Calprotectin

MD: Mediterranean diet; SVD: semi-vegetarian diet; SCD: specific carbohydrate diet; MSCD: modified SCD; SPEN: short-term partial enteral nutrition; WF: whole foods; CD: Crohn’s disease; HLA-B27 and HLA-B7: rat codes.
